# Immunization with *Toxoplasma gondii* peroxiredoxin 1 induces protective immunity against toxoplasmosis in mice

**DOI:** 10.1371/journal.pone.0176324

**Published:** 2017-04-27

**Authors:** Ragab M. Fereig, Yasuhiro Kuroda, Mohamad Alaa Terkawi, Motamed Elsayed Mahmoud, Yoshifumi Nishikawa

**Affiliations:** 1 National Research Center for Protozoan Diseases, Obihiro University of Agriculture and Veterinary Medicine, Inada-cho, Obihiro, Hokkaido, Japan; 2 Department of Animal Medicine, Faculty of Veterinary Medicine, South Valley University, Qena City, Qena, Egypt; 3 Department of Applied Biochemistry, Tokai University, Kita-kaname, Hiratsuka, Kanagawa, Japan; 4 Department of Animal Behavior, Management, Genetics and Breeding, Faculty of Veterinary Medicine, Sohag University, Sohag City, Sohag, Egypt; Institut national de la santé et de la recherche médicale - Institut Cochin, FRANCE

## Abstract

To develop a vaccine against *Toxoplasma gondii*, a vaccine antigen with immune-stimulating activity is required. In the present study, we investigated the immunogenicity and prophylactic potential of *T*. *gondii* peroxiredoxin 1 (TgPrx1). The TgPrx1 was detected in the ascitic fluid of mice 6 days postinfection, while specific antibody levels were low in the sera of chronically infected mice. Treatment of murine peritoneal macrophages with recombinant TgPrx1 triggered IL-12p40 and IL-6 production, but not IL-10 production. In response to TgPrx1, activation of NF-kB and IL-6 production were confirmed in mouse macrophage cell line (RAW 264.7). These results suggest the immune-stimulating potentials of TgPrx1. Immunization of mice with recombinant TgPrx1 stimulated specific antibody production (IgG1 and IgG2c). Moreover, spleen cell proliferation and interferon-gamma production significantly increased in the TgPrx1- sensitized cells from mice immunized with the same antigen. Immunization with TgPrx1 also increased mouse survival and decreased cerebral parasite burden against lethal *T*. *gondii* infection. Thus, our results suggest that TgPrx1 efficiently induces humoral and cellular immune responses and is useful as a new vaccine antigen against toxoplasmosis.

## Introduction

Toxoplasmosis is a heteroxenous zoonotic disease caused by the protozoan parasite *Toxoplasma gondii*, which infects approximately one third of the world population [1]. It also invades almost all endothermic animals. Human infection by *T*. *gondii* is generally not apparent and induces a self-curing disease in immunocompetent individuals. However, the effects of infection are much more severe in immunocompromised patients [2]. Toxoplasmosis in animals, mainly sheep, goats, and pigs, is of great economic importance because it causes abortion, still birth, and neonatal losses. The consumption of food contaminated with the tissue cysts of *T*. *gondii*, such as meat from infected livestock, is the main route of transmission of the parasite to humans [3, 4].

Numerous *Toxoplasma*-derived molecules have been identified as immune-synchronizing effectors that induce dramatic and diverse immunomodulatory pathways. A rhoptry protein of *T*. *gondii*, ROP16, suppresses the expression of the proinflammatory cytokine interleukin 12 (IL-12) by the host macrophages [5]. *Toxoplasma gondii* cyclophilin is reported to enhance macrophage nitric oxide production by binding to C-C chemokine receptor type 5 [6], and its profilin protein has been identified as a critical molecule that stimulates IL-12 production via a Toll-like receptor 11 (TLR11)-dependent pathway [7]. In the same context, the dense granule protein GRA15 of the *T*. *gondii* type II avirulent strain significantly increases the secretion of IL-12 [8].

The peroxiredoxins (Prxs) are a recently described family of antioxidants that are identified in eukaryotes and prokaryotes [9]. Prx acts as an antioxidant enzyme by sweeping hydrogen peroxide and hydroxyl radicals. The catalytic mechanism of the enzyme involves a redox-active cysteine (cys), which is highly conserved in the vicinity of the 47th position of its amino acid sequence [10]. Three Prxs have been identified in *T*. *gondii* tachyzoites, protecting them from oxidative stress: 2-cys Prx1, 1-cys Prx2, and 2-cys Prx3 [11, 12]. Recombinant TgPrx1 promotes an alternative activated macrophage pathway and induces IL-10 secretion via STAT6-dependent and -independent mechanisms, while reducing IL-1β production via caspase 1 [13]. In contrast, *Plasmodium berghei* ANKA Prx strongly induces the macrophage secretion of proinflammatory cytokines, tumor necrosis factor α (TNF-α) and IL-12p40 [14]. In the same way, human Prx1 enhances the production of IL-6 and TNF-α from macrophage cells by binding to TLR4 [15], and induces the secretion of inflammatory IL-23 by activating TLR2 and TLR4 [16].

Macrophages constitute the first line of innate immunity, which contributes to the effective elimination of *T*. *gondii*. This action is mediated by IL-12, which is critical for the endogenous secretion of interferon γ (IFN-γ) [17]. Cellular immune response and IFN-γ production are the most successful strategy for development of potent vaccine candidates because immune protection against *T*. *gondii* in mice is primarily related to Th1 cell mediated immunity and IFN- γ secretion [18, 19]. In addition, several studies unveiled a robust linkage between the canonical signaling pathway of nuclear factor-kappa B (NF-kB) and infection with *T*. *gondii* either by activation or inhibition [20–23]. However, the mechanism of interaction between *T*. *gondii* and NF-kB signaling pathway is still deeply unknown. Although numerous *T*. *gondii* effector molecules were already described as potent immunomodulators, some molecules interact with NF-kB transcription factors or relevant effectors [8, 24–26].

The establishment of novel control and preventive strategies for toxoplasmosis is critical in reducing the risk to public health and livestock production. Currently, the only commercial vaccine (ToxoVax^®^, Intervet B.V.), based on live attenuated tachyzoites of *T*. *gondii* strain S48, is available for veterinary use in a limited number of countries to minimize the incidence of abortion in sheep [27, 28]. This vaccine has certain limitations and cannot be used in humans because live vaccines can potentially recover their virulence and induce infection [29]. Moreover, most available drugs used for the treatment and control of toxoplasmosis are only effective in acute case, whereas others, such as sulfadoxine/pyremethamine, have highly toxic effects on the treated individuals, including teratogenic effects and cutaneous lesions [30, 31]. Therefore, the development of an effective and safe vaccine against *T*. *gondii* would be extremely valuable in controlling this parasitic infection in humans and animals.

The molecular and biochemical properties of the TgPrx1 have been extensively investigated. TgPrx1 is expressed in the cytosol and protects cells against the free radicals generated as the byproducts of vital processes that occur in the cytoplasm [12, 32]. Moreover, alignment of amino acid sequence between TgPrx1 and those of many other living creatures indicates the high specificity of TgPrx1, suggesting that TgPrx1 is a potent vaccine antigen and a candidate of drug target [33]. Therefore, in the present study, we investigated the immunological and protective potentials of TgPrx1. Only one study has discussed relevant research into TgPrx1 [13], and no study has yet clarified the immunoprophylactic potential of this antigen.

## Materials and methods

### Ethics statement

In this study, we strictly followed the recommendations of the Guide for the Care and Use of Laboratory Animals of the Ministry of Education, Culture, Sports, Science and Technology, Japan. The protocol was approved by the Committee on the Ethics of Animal Experiments at the Obihiro University of Agriculture and Veterinary Medicine (permission numbers 25–66, 26–68, 27–30, 28–49). All painful experimental treatments and surgical operations were implemented under general anesthesia induced with isoflurane.

### Animals

Female C57BL/6J mice aged 6–7 weeks were purchased from Clea Japan (Tokyo, Japan) and allocated to the immunomodulatory or immunization experiments. Seven-week-old female BALB/c mice and female white Japanese rabbits were obtained from Clea Japan for the preparation of polyclonal antibodies against recombinant protein TgPrx1. All the animals used in the study were treated under the guiding principles for the care and use of research animals promulgated by Obihiro University of Agriculture and Veterinary Medicine.

### Parasites and cell cultures

*Toxoplasma gondii* PLK (avirulent type II strain) and RH (virulent type I strain) were used in this study. The parasites were maintained in Vero cells (African green monkey kidney epithelial cells) cultured in Eagle’s minimum essential medium (EMEM; Sigma, St Louis, MO, USA) supplemented with 8% heat-inactivated fetal bovine serum (FBS; Nichirei Biosciences, Tokyo, Japan) and 1% streptomycin–penicillin (Sigma). For the purification of tachyzoites, the parasites and host cell debris were washed with sterile phosphate-buffered saline (PBS) and the infected cell monolayer was peeled from the plate with a cell scraper (BD Bioscience, San Jose, CA, USA). The final cell pellet was resuspended in RPMI 1640 medium (Sigma) and passed through a 27-gauge needle and a filter with a pore size of 5.0 μm (Millipore, Bedford, MA, USA).

### Expression and purification of recombinant proteins

**Fig 1:**
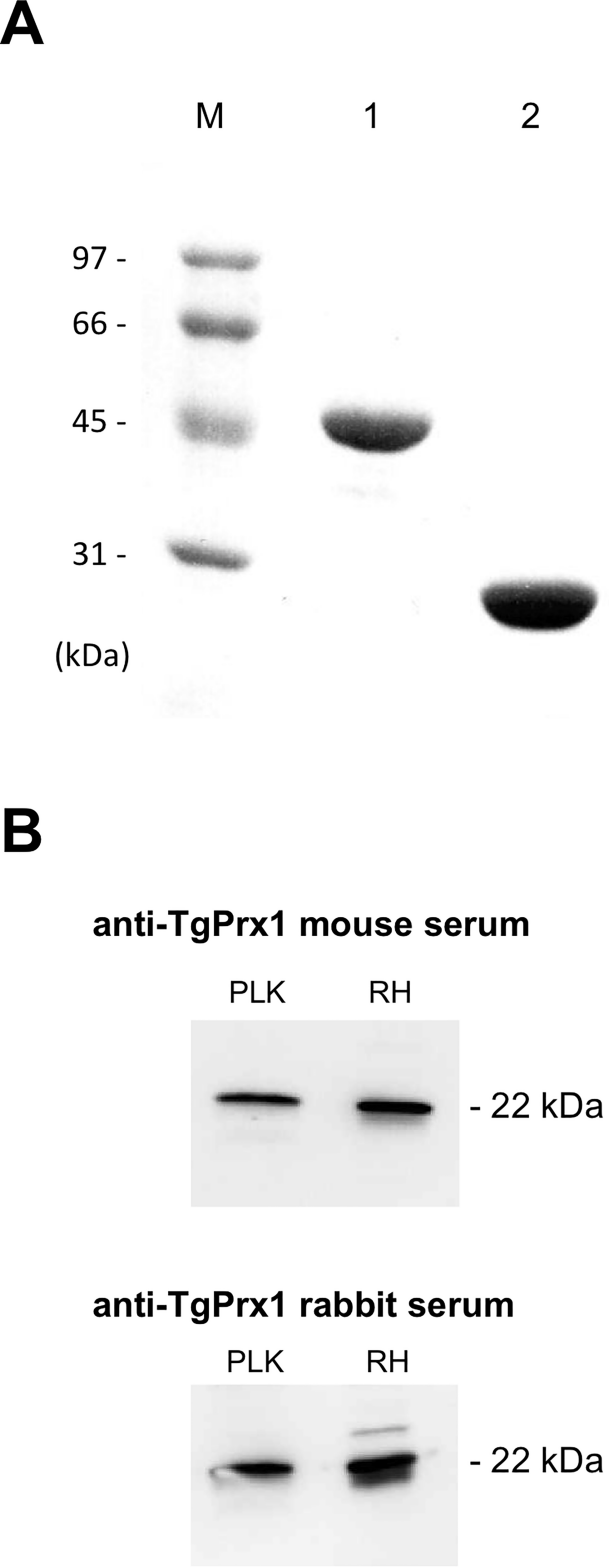
Expression of recombinant proteins and detection of TgPrx1 using specific antibodies. (A) Sodium dodecyl sulfate polyacrylamide gel electrophoresis (SDS-PAGE) of recombinant proteins, with Coomassie Blue staining. Lanes: M, molecular mass marker; lane 1, TgPrx1–GST; lane 2, GST. (B) Western blot using lysate of *T*. *gondii* tachyzoites (RH and PLK strains) using mouse and rabbit anti-TgPrx1 sera.

The *TgPrx1* gene (GenBank accession number, XM_002371315.1) was amplified from cDNA of *T*. *gondii* PLK strain with PCR using oligonucleotide primers that included a *Bam*HI site (underlined) in the forward primer 5′-TA GGA TCC ATG CCG GCC CCG ATG GTG TCT-3′ and an *Xho*I site (underlined) in the reverse primer 5′-AG CTC GAG TTA CTT GCT TCC GAG ATA CTC-3′. The PCR products digested with Bam*HI* and *Xho*I, were inserted into the pGEX-4T3 plasmid vector treated with the same restriction enzymes (Amersham Pharmacia Biotech, Madison, CA, USA). Recombinant TgPrx1 was expressed as glutathione *S*-transferase (GST) fusion protein (TgPrx1-GST) in *Escherichia coli* BL21(DE3) (New England BioLabs Inc., Ipswich, MA, USA). The expression achieved at 37 C for 8 h after induction with 1 mM isopropyl β-d-1-thiogalactopyranoside (Wako Inc., Osaka, Japan). The resulting *E*. *coli* cells were harvested in TNE buffer (100 mM Tris-HCl [pH 8], 100 mM NaCl, 5 mM EDTA) with high-speed centrifugation (10,000 × *g*, 4°C, 30 min), lysed with 1% Triton in PBS and 50 mg/mL lysozyme, sonicated on ice, and then centrifuged as in the previous step. The supernatant was purified with Glutathione Sepharose 4B beads (GE Healthcare Life Sciences), according to the manufacturer’s instructions. In brief, the supernatant–beads mixture was incubated overnight at 4°C with rotation, and the GST-fused protein was eluted with elution buffer (100 mM Tris-HCl [pH 8], 100 mM NaCl, 5 mM EDTA, 20 mM reduced glutathione powder; Wako Inc.). The yield of protein was dialyzed in PBS and the endotoxins were removed with a Detoxi-Gel Endotoxin Removing Column (Thermo Scientific, Waltham, MA, USA). For use in cell culture, the proteins were filtered with a 0.45-μm low-protein binding Supor^®^ membrane (Pall Life Sciences, Ann Arbor, MI, USA). The endotoxin levels in the TgPrx1-GST and GST preparations were estimated with Limulus Amebocyte Lysate reagents (Seikagaku Inc., Tokyo, Japan), and no endotoxin was detected in the tested protein lots. The purity and quantities of the proteins were determined as a single band on sodium dodecyl sulfate-polyacrylamide gel electrophoresis (SDS-PAGE) followed by staining with Coomassie Brilliant Blue R250 (MP Biomedicals Inc., Illkirch-Graffenstaden, France). The protein concentrations were measured with a bicinchoninic acid (BCA) protein assay kit (Thermo Fisher Scientific, Inc., Rockford, IL, USA). The recombinant TgPrx1–GST and GST proteins were isolated with apparent molecular weights of 49 kDa and 26 kDa, respectively, which were consistent with the expected molecular size of each protein (Fig 1).

### Generation of polyclonal antibodies against TgPrx1 and purification of immunoglobulin G (IgG)

Seven-week-old female BALB/c mice were intraperitoneally injected on day 0 with 100 μg of purified recombinant protein TgPrx1-GST emulsified in Freund’s complete adjuvant (Sigma). The same protein in Freund’s incomplete adjuvant (Sigma) was injected into the mice on days 14, 28, and 42 after the first immunization. Sera were collected from the tail veins of the mice at −2, 12, 26, and 40 days and with heart puncture after 49 days. Sera from the inoculated mice that displayed the highest sensitivity to and specificity for *T*. *gondii* were used in subsequent experiments. Purified recombinant protein (1 mg) was also subcutaneously injected into a female Japanese white rabbit. The presacrifice serum was collected from the ear vein. The sacrifice serum sample was collected after the antibody titers were measured. The titer of IgG was estimated with an indirect enzyme-linked immunosorbent assay (ELISA), with the method discussed below. IgG was purified from 2 mL of the rabbit serum using protein A chromatography columns (Bio-Rad Laboratories, Hercules, CA, USA), according to the manufacturer’s instructions. Aliquots of IgG were checked for protein purity and quantity with SDS-PAGE. The protein concentration of the purified rabbit polyclonal IgG were measured with a BCA protein assay kit.

### Western blot analysis

The protein lysates from purified *T*. *gondii* tachyzoites (15 μg/10 μl) were mixed with 10 μl of 2×SDS gel reducing loading buffer (62.5 mM Tris-HCl pH 6.8, 2% (w/v) SDS, 140 mM 2-mercaptoethanol, 10% (w/v) glycerol and 0.02% (w/v) bromophenol blue). Samples were heated at 95°C for 5 min and separated on a 15% polyacrylamide gel. After SDS polyacrylamide gel electrophoresis, the protein bands in the gel were transferred to a nitrocellulose membrane (Whatman GmbH, Dassel, Germany). After washing twice with PBS containing 0.05% (v/v) Tween 20 (PBS-T), the membranes were blocked with PBS containing 3% (w/v) skimmed milk (PBS-SM) for 12 h at 4°C. After two further washes, the membranes were incubated with anti-TgPrx1 mouse or rabbit serum (1:200) for 1 h at room temperature. After washing three times, the membranes were incubated with horseradish peroxidase-conjugated immunoglobulin G (1:2,000; Amersham Pharmacia Biotech, Piscataway, NJ, USA) diluted in PBS-SM, for 1 h at 37°C. After washing five times, the proteins were visualized using ECL™ western blotting detection reagents (GE Healthcare UK Ltd., Buckinghamshire, UK) by VersaDoc™ imaging system (Nippon Bio-Rad Laboratories, Tokyo, Japan) according to the manufacturer’s recommendations. To confirm the reactivity of anti-TgPrx1 mouse or rabbit serum, western blot using TLA was performed. As shown in Fig 1B, protein with apparent molecular weights of 22 kDa was detected from the TLA of PLK and RH strains, which were consistent with the expected molecular size of TgPrx1 [11].

### Measuring the levels of secreted antigen using double antibody sandwich (DAS)-ELISA

Rabbit IgG directed against TgPrx1 was diluted to 10 μg/mL in 0.05 M carbonate buffer (pH 9.6). IgG (0.5 μg per well) was used as the capture antibody to coat microtiter plates (Nunc, Roskilde, Denmark) at 4°C overnight. Blocking solution (200 μL; 3% skimmed milk in PBS) was added to each well and the plates were incubated at 37°C for 2 h. After the addition of the test samples in duplicate and the recombinant protein as the standard, the plates were incubated at 37°C for 30 min. The test samples were collected from the peritoneal fluid of C57BL/6 mice infected with the RH or PLK *T*. *gondii* strain. The plates were washed six times with washing solution (0.05% Tween 20 in PBS). Anti-TgPrx1 mouse serum, diluted at 1:3000 in blocking solution, was added to each well and the plate was incubated at 37°C for 1 h. After six more washes, the plates were incubated with horseradish peroxidase (HRP)-conjugated anti-mouse IgG antibody, diluted 1:5,000 in blocking solution, at 37°C. Substrate solution (100 μL; 0.1 M citric acid, 0.2 M sodium phosphate, 0.003% H_2_O_2_, 0.3 mg/mL 2,2′-azino-bis[3-ethylbenzothiazoline-6-sulphonic acid]; Sigma) was added to each well to visualize binding. The absorbance at 415 nm was measured with an ELISA reader (Corona MTP-120 microplate reader; Corona, Tokyo, Japan). The concentration of TgPrx1 antigen was calculated for each sample by standardization against the purified recombinant protein.

### Indirect ELISA to detect TgPrx1- specific antibody

Recombinant TgPrx1-GST and GST proteins were adjusted to concentrations of 0.1 μM with coating buffer, added to the wells of ELISA plates, and incubated overnight at 4°C. Recombinant antigen of TgGRA7 was also used as a reference antigen [34]. The plates were washed twice with washing buffer and then blocked with PBS containing 3% skim milk (PBS-SM) for 1 h at 37°C. The plates were washed twice and 50 μL of the test serum, or the positive or negative control serum, diluted 1:100 with PBS-SM, was added to duplicate wells. The plates were incubated at 37°C for 1 h. After the plates were washed six times, they were incubated with HRP-conjugated goat anti-mouse total IgG, IgG1 or IgG2c antibody (Bethyl Laboratories, Montgomery, TX, USA), diluted 1:4,000 with PBS-SM, at 37°C for 1 h. The plates were washed six times, and 100 μL of substrate solution was added to each well. After the samples were incubated at room temperature in the dark for 1 h, the absorbance at 415 nm was determined with an ELISA reader. The ELISA results for TgPrx1-specific antibody were determined by measuring the mean optical density of GST protein subtracted from that of TgPrx1-GST antigen. The formulations for the coating and washing buffers and the substrate solution were as described in the DAS-ELISA experiment.

### Preparation and culture of murine peritoneal macrophages

Four days after C57BL/6J mice were injected intraperitoneally with 2 mL of 4.05% BBL™ Brewer modified thioglycolate medium (Becton Dickinson, Sparks, MD, USA), their peritoneal macrophages were collected with two peritoneal lavages of 5 mL of cold PBS. The harvested cells were centrifuged at 1,300 × *g* for 10 min and suspended in Dulbecco’s modified Eagle’s medium (DMEM; Sigma) containing 8% FBS. Red blood cells were removed with lysis buffer (0.83% NH_4_Cl, 0.01 M Tris-HCl [pH 7.2]) and then washed away with medium. The macrophage suspension was added to a 96-well microplate at 3 × 10^5^ cells/well and incubated at 37°C in a 5% CO_2_ incubator for 4 h, allowing the cells to settle to the bottom. The wells were washed with FBS-free DMEM to remove the floating cells, and incubated for 20 h after the addition of the indicated stimulants, including positive and negative controls. To confirm the effects of the resident lipopolysaccharide (LPS) in the protein samples, polymixin B (Sigma) was used.

### Culturing and stimulation of RAW 264.7 cell lines

The mouse macrophage RAW 264.7 was provided by the RIKEN BRC through the National Bio-Resource Project of the MEXT, Japan. NF-kB secreted alkaline phosphatase reporter cell line generated from RAW 264.7 (NF-kB/SEAP cells) was obtained from Novus biological inc. (Littleton, CO, USA). The cells were cultured in DMEM supplemented with 10% (v/v) fetal bovine serum and 100 U/mL penicillin, 100 mg/mL streptomycin (Life Technologies, Darmstadt) for both cell lines while 0.5 mg/mL G418 (Geneticin, Roche, Mannheim, Germany) was added for NF-kB/SEAP cells for drug based-selection and then cells were cultivated at 37°C in a 5% CO_2_ incubator. For induction experiments, cells were seeded in 96-well plate (2x10^5^ in 100 μL/well) and incubated at 37°C for 4 hrs to allow adherence of the cells to the bottom. The proteins of TgPrx1-GST and GST at final concentrations 10 nM and 100 nM, and LPS at a final concentration 10 ng/mL as a positive control and only medium as a negative control were added to the cells, then the plates were incubated again at 37°C for 48 hrs. The stimulants including recombinant proteins, LPS and the medium were added to the cells with and without polymixin B at a final concentration 20 μg/mL to validate the test against the effect of resident endotoxin. Optimum condition including the concentrations for recombinant proteins, LPS and polymixin B, incubation period and RAW cell density were adjusted according to several preliminary experiments.

### Preparation of *Toxoplasma* lysate antigens (TLA)

*Toxoplasma gondii* lysate antigens were prepared from tachyzoites of PLK and RH strains as previously described [35], with slight modifications. Briefly, the purified tachyzoites were washed with PBS and adjusted to 1×10^8^ tachyzoites/mL of PBS. The parasites were destroyed by sonication on ice followed with exposure to three freezing–thawing cycles. The crude extract was harvested after centrifugation at 10,000 × *g* for 10 min and filtration with a 0.45-μm low-protein binding Supor® membrane. The concentration was measured using a BCA protein assay kit.

### Stimulation of splenocytes for measuring the proliferation assay and cytokines production

Spleens of immunized mice were surgically dissected under aseptic condition after 2 weeks of the last immunization and processed as illustrated previously [36], with slight modifications. Spleen of each mouse was crushed between 2 sterile glass slides, then cells were suspended thoroughly in RPMI 1640 medium (Sigma) supplemented with 10% fetal bovine serum. Red blood cells were destructed via addition of lysis buffer (0.83% NH_4_Cl and 0.01 M Tris-HCl, pH 7.2) to the cell suspension then washed with medium. The cells were plated into 96-well microplates at 2.5 x 10^5^/100 μl/well in RPMI 1640 medium. Spleen cells were stimulated with TgPrx1-GST and GST recombinant proteins, TLA, concanavalin A (ConA; Sigma-Aldrich, St Louis, MO) as positive control and stimulant-free medium as a negative control. The plates were incubated for 48 h at 37°C in 5% CO_2,_ then 100 μl from the supernatants of cultures were collected and assayed for cytokines. Simultaneously, for estimation of splenocyte proliferation assay, 10 μl of Cell Counting Kit-8 reagent (CCK-8, Dojindo Laboratories, Kumamoto, Japan) was added to each well for the previously stimulated cells. After 2 h of incubation at 37°C in 5% CO_2_, optical density was measured using an ELISA reader at 450 nm.

### Sandwich ELISA to measure cytokine levels and secreted alkaline phosphatase (SEAP) reporter assay

The supernatant was collected from the cell culture of mouse peritoneal macrophages, RAW 264.7, or spleen cells to measure the levels of cytokines. The cytokines (IL-4, IL-6, IL-10, IL-12p40 and IFN-γ) were measured with commercial ELISAs (Pierce Biotechnology Inc., Rockford, IL, USA), according to the manufacturer’s recommendations. The cytokine concentrations were calculated from standard cytokine curves constructed from samples run on the same plate. The culture supernatant from NF-kB/SEAP cells was collected to measure the levels of SEAP by SEAP reporter assay kit (Novus) according to the manufacturer’s instructions. The SEAP concentrations were calculated from SEAP assay standard curve generated by adding a serial dilution of SEAP protein run on the same plate and performed according to the kit assay protocol.

### Immunization and infection

To investigate the immunoprophylactic properties of TgPrx1, mice were inoculated subcutaneously with 25 pmol of recombinant TgPrx1-GST or GST protein in PBS, or with PBS alone (each 100 μL) three times at 14-day intervals (Total number = 18 mice per group from 3 independent trials). Fourteen days after the third immunization, the mice were challenged intraperitoneally with 1 × 10^3^ tachyzoites of the *T*. *gondii* PLK strain. The mouse survival rates were measured for 30 days after challenge. Serum (20 μL) was collected from the mice at 14, 28, and 42 days after immunization, via the tail vein, to measure the specific antibodies directed against TgPrx1 with ELISAs. To confirm the lack of an antibody response in the unvaccinated and uninfected mice, control sera were collected from all the animals on day 2 before immunization. Thirty days after infection, serum and brain samples were collected from all the surviving mice after they were euthanized.

### DNA isolation and quantitative PCR analysis

The parasites in the brains of the immunized mice were extracted, purified, and quantified as previously described [37], with slight modifications. Brain DNA was extracted by incubation with extraction buffer (0.1 M Tris-HCl [pH 9.9], 1% SDS, 0.1 M NaCl, 1 mM EDTA, 1 mg/mL proteinase K) at 55°C. Phenol–chloroform extraction and ethanol precipitation were used to purify the DNA. The parasite DNA was amplified with primers specific for the *T*. *gondii* B1 gene (5′-AAC GGG CGA GTA GCA CCT GAG GAG-3′ and 5′-TGG GTC TAC GTC GAT GGC ATG ACA AC-3′), which have already been shown to detect all known parasite strains [38]. The PCR mixture (25 μl total volume) contained 1 × SYBR Green PCR buffer, 2 mM MgCl_2_, 200 μM each deoxynucleoside triphosphate (dATP, dCTP, and dGTP), 400 μM dUTP, 0.625 U of AmpliTaq Gold DNA polymerase, 0.25 U of AmpErase Uracil-*N*-Glycosylase (AB Applied Biosystems, Carlsbad, CA, USA), 0.5 μmol of each primer, and 50 ng of genomic DNA. Amplification was performed with a standard protocol recommended by the manufacturer (AB Applied Biosystems, 2 min at 50°C, 10 min at 95°C, 40 cycles at 95°C for 15 s, and 60°C for 1 min). Amplification, data acquisition, and data analysis were performed with the ABI Prism 7900HT Sequence Detection System (Applied Biosystems), and the calculated cycle threshold (Ct) values were exported to Microsoft Excel for analysis. A standard curve was established with *T*. *gondii* DNA extracted from 1 × 10^5^ parasites using 1 μL of a serial dilution ranging from 10,000 to 0.01 parasites. The parasite numbers were calculated by interpolation on the standard curve, with the Ct values plotted against a known concentration of parasite. After amplification, the melting-curve data for the PCR products were acquired using stepwise increases in temperature from 60°C to 95°C. The data were analyzed using Dissociation Curves version 1.0 F (AB Applied Biosystems).

### Statistical analysis

The GraphPad Prism 5 software (GraphPad Software Inc., La Jolla, CA, USA) was used. Data are presented as means ± standard deviation. Statistical analyses were performed with the Student’s *t* test, one-way or two-way analysis of variance (ANOVA) followed by the Tukey–Kramer test for group comparisons. The significance of the differences in survival was analyzed with a χ^2^ test. The levels of statistical significance are presented with asterisks or letters and are defined in each figure legend, together with the name of the statistical test used. A *P* value < 0.05 was considered statistically significant.

## Results

### Detection of TgPrx1 antigen and specific antibody during *T*. *gondii* infection

**Fig 2:**
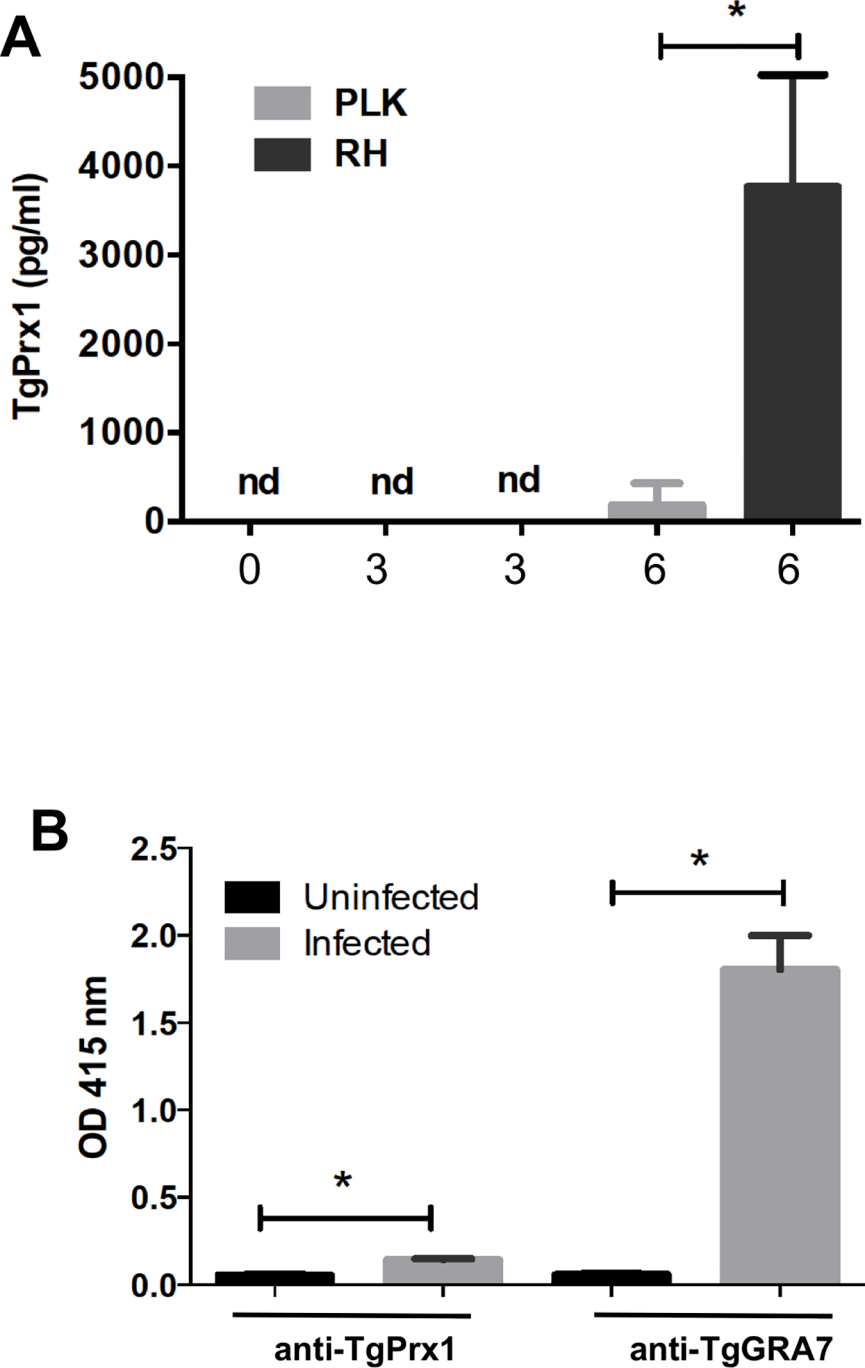
Antigen and antibody levels of TgPrx1 in body fluids of infected mice. (A) C57BL/6 mice (n = 4) were intraperitoneally infected with 10^3^ *T*. *gondii* PLK or RH tachyzoites. TgPrx1 antigen was measured in ascitic fluid collected from experimentally infected mice at 0, 3, and 6 days postinfections (dpi). Each value represents the mean ± standard deviation of quadruplicate samples. nd, not detected. *, statistically significant differences were observed between the two groups with the Student’s *t* test (*P* < 0.05). **(B)** Production of IgG antibodies against TgPrx1 in chronically infected mice. C57BL/6 mice (n = 5) were intraperitoneally infected with 10^3^ *T*. *gondii* PLK tachyzoites. Serum samples were collected from the mice 4 weeks after infection and tested with indirect ELISAs, using recombinant TgPrx1-GST and TgGRA7 antigens. The mean optical density (OD) was determined at a wavelength of 415 nm. Each bar represents the mean ± standard deviation. Sera of uninfected mice (n = 4) were used as the negative control. *, statistically significant differences were observed between the uninfected and infected mice with the Student’s *t* test (*P* < 0.05).

The peritoneal fluid of mice experimentally infected with *T*. *gondii* PLK (avirulent type II strain) or RH (virulent type I strain) were examined to detect the release of TgPrx1 antigen into the body fluid during infection (Fig 2A). Although the release of TgPrx1 into the peritoneal fluid of both the RH- and PLK-infected mice was confirmed, higher level of secreted antigen was detected in the fluid of the RH-infected animals. To determine the efficacy of TgPrx1 for initiating the production of specific antibodies, the sera of mice infected with PLK, collected 4 weeks after infection were tested with an indirect ELISA based on the TgPrx1 antigen and on TgGRA7, a well-known potent diagnostic antigen [34]. Although TgPrx1-specific antibodies were detected, the levels of the antibodies were significantly lower than the levels of TgGRA7-specific antibodies (Fig 2B). These results indicate that TgPrx1 is less antigenic than TgGRA7 while they are released into the body fluid of mice after *T*. *gondii* infection.

### Activation of murine macrophages by TgPrx1

**Fig 3:**
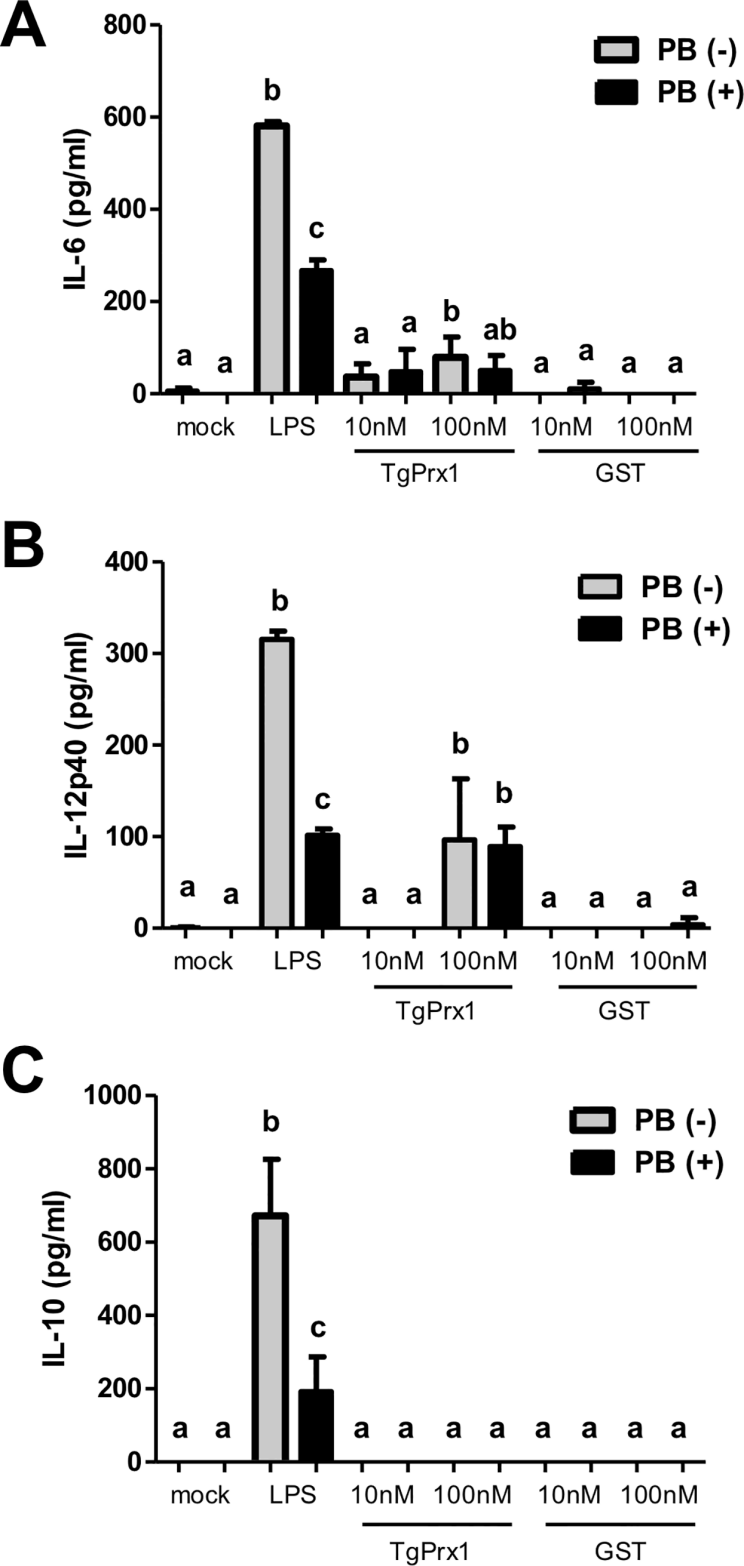
Production of IL-6, IL-12p40 and IL-10 by murine peritoneal macrophages. Murine peritoneal macrophages were treated with 1 ng/mL LPS and recombinant TgPrx1-GST or GST protein for 20 h in the presence or absence of 1 μg/mL polymixin B. The levels of IL-6 (A), IL-12p40 (B) and IL-10 (C) value represents the mean ± standard deviation of quadruple samples. The results are representative of three repeated experiments with similar results. The different letters above the bars in the graphs indicate statistically significant differences among the test groups and the mock group (one-way ANOVA plus Tukey–Kramer *post hoc* analysis, *P* < 0.05).

To investigate whether the released TgPrx1 stimulated immune cells, we stimulated murine peritoneal macrophages with the recombinant TgPrx1-GST in vitro (Fig 3). TgPrx1-GST treatment triggered IL-6 production while there was no significant difference between the TgPrx1-GST and mock-treated macrophages (Fig 3A). Furthermore, TgPrx1-GST at concentrations of 100 nM significantly enhanced the production of IL-12p40 compared with that expressed in mock-treated cells (Fig 3B). On the contrary, anti-inflammatory cytokine IL-10 was not detected in the culture of macrophages treated with TgPrx1-GST while LPS stimulation triggered IL-10 production (Fig 3C). The levels of IL-6 and IL-12p40 after treatment with TgPrx1 were not altered by the presence of polymixin B. Treatment of the cells with LPS also triggered the expression of these cytokines, but polymixin B reduced their expression. GST and the mock treatment did not trigger any response. These results demonstrate the capacity of TgPrx1 to enhance macrophage responses.

**Fig 4:**
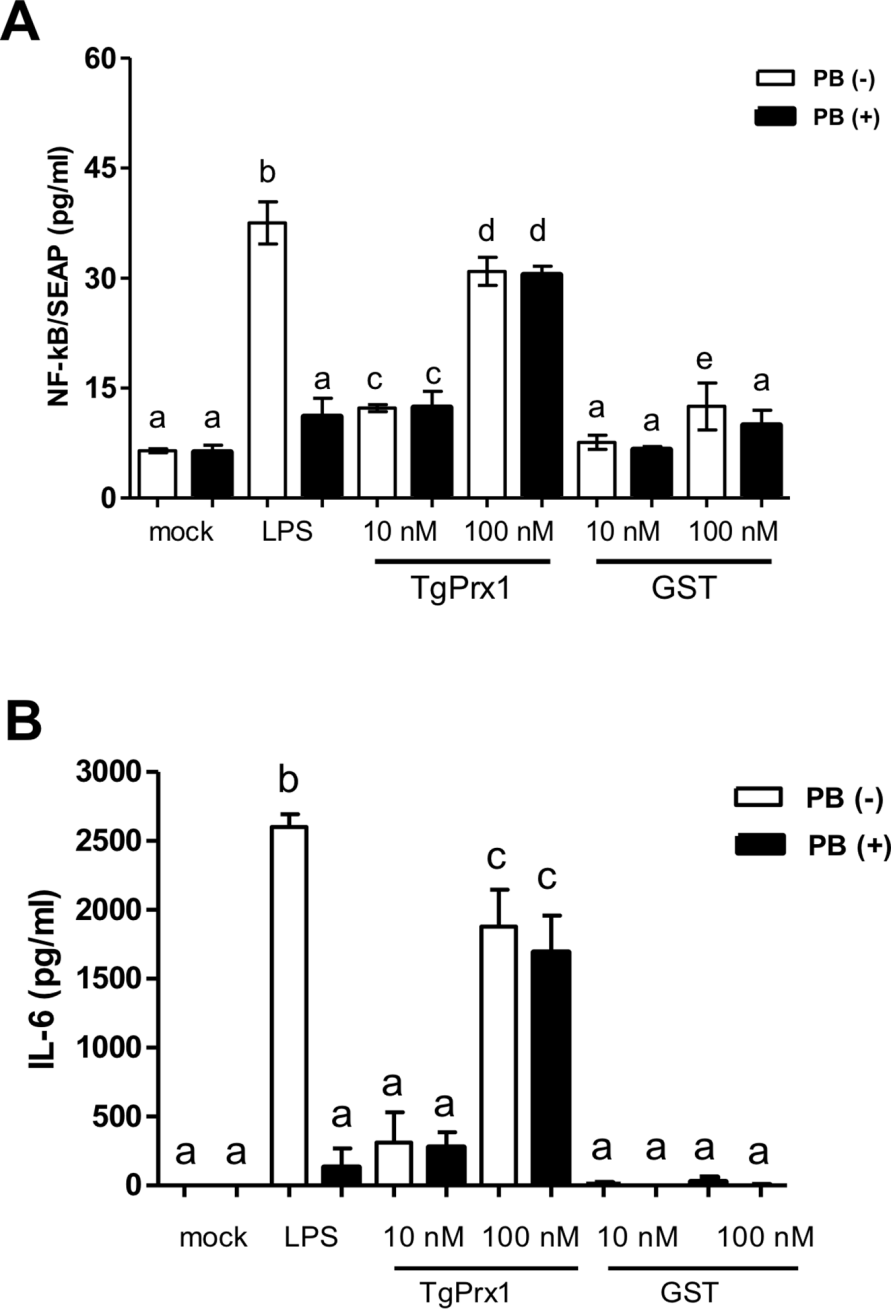
Effects of recombinant TgPrx1 on RAW cell lines. NF-kB/SEAP cells (A) and RAW 264.7 cells (B) were treated with 10 ng/mL LPS and recombinant TgPrx1-GST or GST protein for 48 h in the presence or absence of 20 μg/mL polymixin B to measure the SEAP and IL-6, respectively. Each value represents the mean ± standard deviation of quadruple samples. The different letters above the bars in the graphs indicate statistically significant differences among the test groups and the mock group (one-way ANOVA plus Tukey–Kramer *post hoc* analysis, *P* < 0.05).

The immunogenicity of TgPrx1 was also estimated by using RAW cells. The TgPrx1-GST induced significant secretion of NF-kB dependent SEAP from NF-kB/SEAP cells (Fig 4A). The production of SEAP was evident at concentration of 10 nM and the production was clearly observed at the concentration of 100 nM which was comparable to the positive control level induced with LPS. Addition of polymixin B induced obvious reduction in the production of SEAP for LPS-treated cells, but not TgPrx1-GST-treated cells, indicating the efficacy of polymixin B and the genuine stimulation by recombinant protein of TgPrx1. In the same context, TgPrx1-GST enhanced the production of IL-6 from RAW 264.7 cells (Fig 4B). Although we measured the level of IL-12p40 in RAW 264.7 cells, the level was undetectable even in the positive control samples using LPS. Collectively, recombinant TgPrx1 exhibited a potential effect in promoting the function of murine macrophages via NF-kB signaling pathway.

### Immunization of mice with TgPrx1 against *T*. *gondii* infection

**Fig 5:**
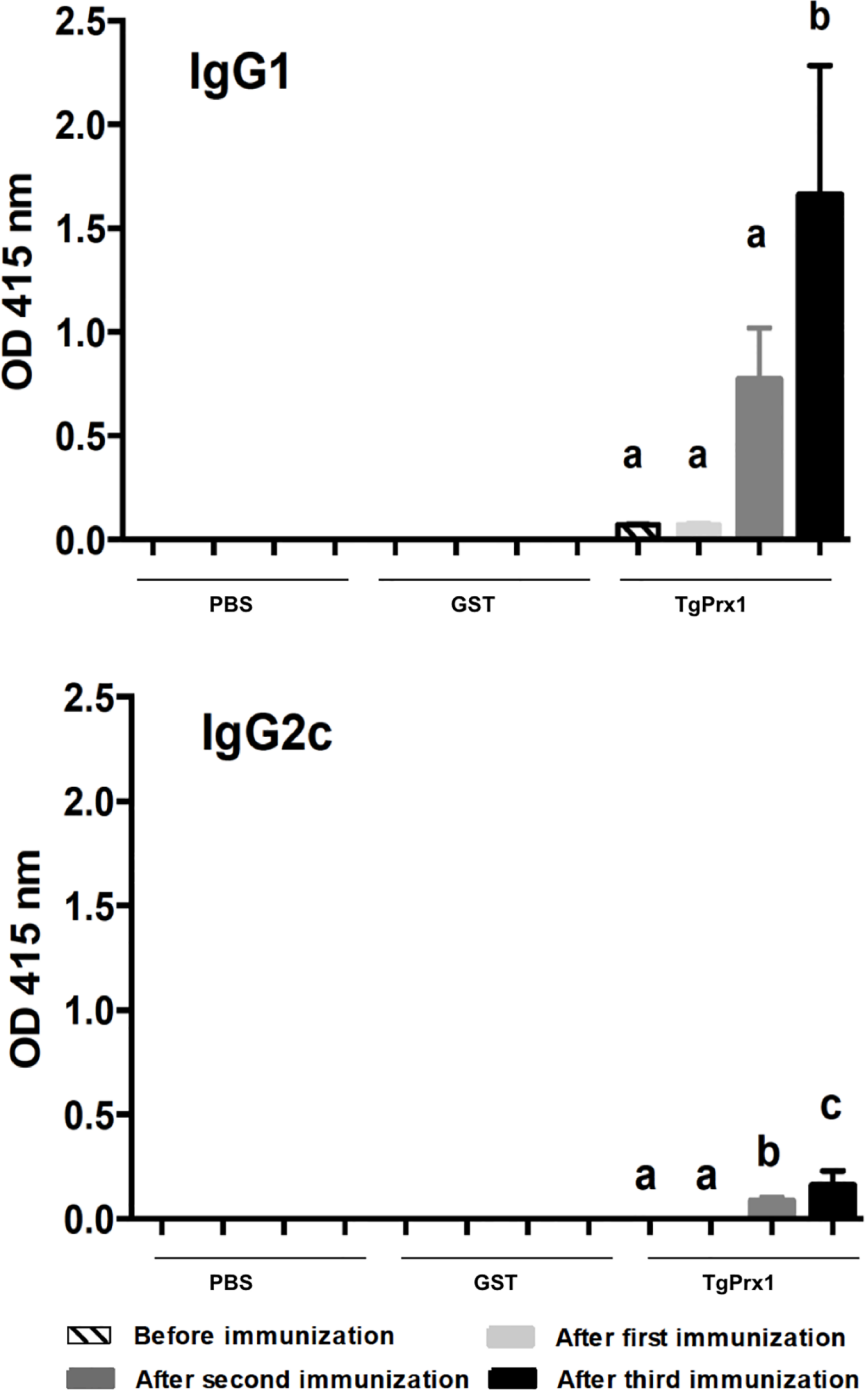
Production of specific antibodies against TgPrx1. C57BL/6 mice were subcutaneously immunized with 25 pmol of recombinant protein (GST and TgPrx1-GST) or PBS (control group). Sera were collected from all mouse groups 2 days before immunization and tested for specific IgG1 and IgG2c antibodies with indirect ELISAs. The antibody responses of each experimental group were tested against the recombinant proteins: TgPrx1-GST, and GST. The mean optical density (OD) was determined at a wavelength of 415 nm. The readings for the GST protein were subtracted from those of the TgPrx1-GST antigen. Each bar represents the mean ± standard deviation for the mice used per group (n = 6) and the results are representative of three independent experiments with similar results. The different letters above the bars in the graphs indicate statistically significant differences among the same immunization group (one-way ANOVA plus Tukey–Kramer *post hoc* analysis, *P* < 0.05).

**Fig 6:**
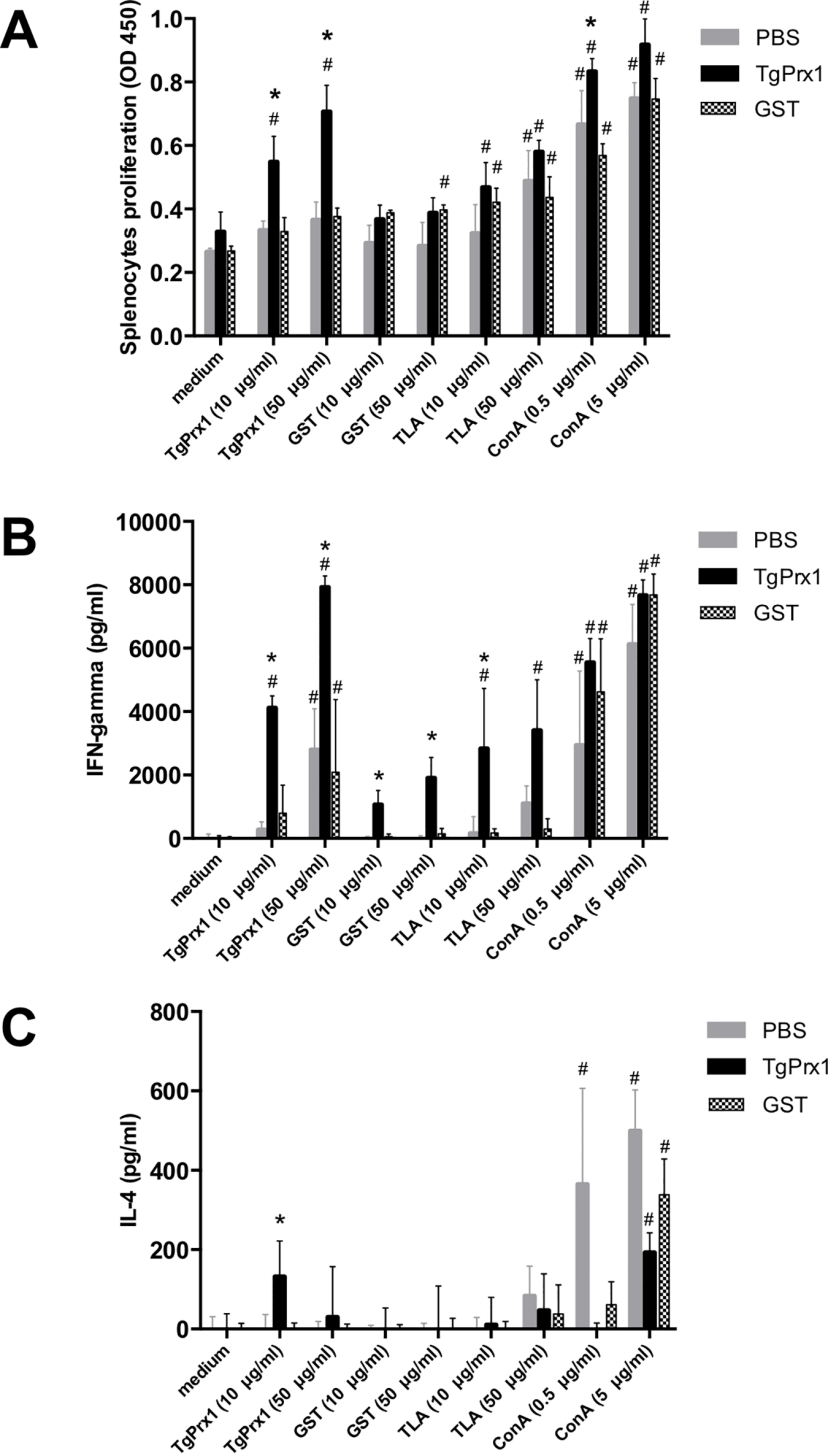
Proliferation and cytokine production of spleen cells. Single-cell suspensions were prepared from the spleens of individual mice immunized with PBS, TgPrx1-GST, or GST and cultured for 48 h in the presence of Con A, TLA, TgPrx1-GST, GST, or without any stimulator (Medium). (A) Cell proliferation was measured at 48 h. (B) The culture supernatants were assayed for IFN-γ, and IL-4 production with ELISAs. Each bar represents the mean ± standard deviation (PBS; n = 3, TgPrx1 and GST; n = 4). *, statistically significant differences were observed between TgPrx1-GST-immunized group and other groups in same stimulator with two-way ANOVA and Tukey–Kramer *post hoc* analysis, *P* < 0.05. **#**, statistically significant differences were against medium well in same immunized group with two-way ANOVA and Tukey–Kramer *post hoc* analysis, *P* < 0.05.

Because the recombinant TgPrx1 protein stimulated the macrophage response, we evaluated its utility as a vaccine antigen with immune-stimulating activity to control *T*. *gondii* infection. As shown in Fig 5, mice immunized with TgPrx1-GST produced significant levels of specific antibodies, including IgG1 and IgG2c antibodies. The levels of antibodies developed against TgPrx1 were significantly higher than in the control mice inoculated with PBS or GST. To determine whether immunization with TgPrx1-GST induced the cellular immune response, the proliferation of spleen cells and the production of IFN-γ and IL-4 were measured following the *in vitro* stimulation of spleen cells isolated from mice after the third immunization (Fig 6). The proliferation of spleen cells was enhanced in TgPrx1-GST-immunized mice by stimulation with TgPrx1-GST at 10 and 50 μg/ml (Fig 6A). The production of IFN-γ was higher in the spleen cells from mice immunized with TgPrx1-GST than in the cells from animals immunized with PBS or GST when they were stimulated with TLA or TgPrx1-GST (Fig 6B). Interestingly, treatment of spleen cells from mice immunized with PBS or GST with TgPrx1-GST triggered IFN-γ production compared with no treated cells (Fig 6B). However, the IL-4 production in the spleen cells from mice immunized with TgPrx1-GST and the control mice inoculated with PBS or GST was not significantly enhanced by stimulation with TLA or immunized antigens, except for the treatment of spleen cells from TgPrx1-GST-immunized mice with 10 μg/ml TgPrx1-GST (Fig 6C). Together, these results indicate that immunization with TgPrx1-GST triggered parasite- and antigen-specific humoral and cell-mediated immune responses in the mice.

**Fig 7:**
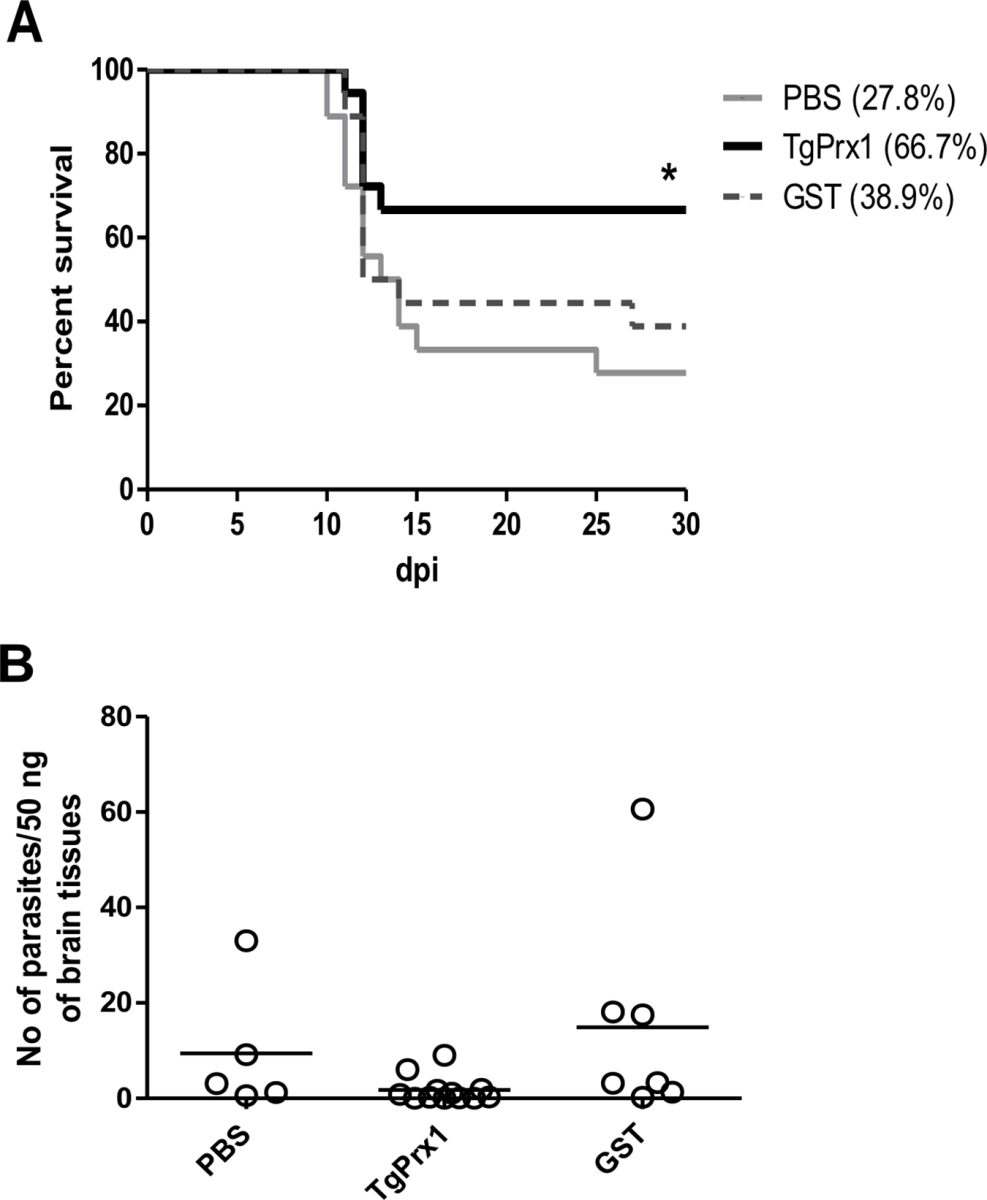
Survival of mice and parasite numbers in brains of surviving mice. (A) Six mice per group were immunized with TgPrx1-GST, GST, or PBS, and then challenged with 1 × 10^3^ tachyzoites of the *T*. *gondii* PLK strain. The survival rates (surviving mice/total mice) are calculated from three pooled independent experiments: PBS, 5/18 (27.8%); TgPrx1, 12/18 (66.7%); GST, 7/18 (38.9%). *, statistically significant differences in the survival rates at 30 days postinfection (dpi) were observed between the PBS-injected group and the recombinant-protein-immunized groups with a χ^2^ test (*P* < 0.05). (B) Parasite numbers in the brains of the surviving mice at 30 dpi. Results are from three pooled independent experiments (PBS, n = 5; TgPrx1, n = 12; GST, n = 7). The results were analyzed with one-way ANOVA plus a Tukey–Kramer *post hoc* analysis, but there were no significant differences.

The survival rate of mice immunized with TgPrx1-GST was 66.7% that was markedly higher than those of the GST- (38.9%) and PBS-injected (27.8%) mice used as the controls (Fig 7A). For more confirmation of the protective efficacy of the TgPrx1 in the immunized mice, the brains of the surviving mice in all the groups were collected 30 days after infection to quantify the parasite with quantitative PCR. The number of parasites in the TgPrx1-GST- immunized groups was lower than those in the GST- and PBS-injected groups, although the differences were not statistically significant (Fig 7B.). This result indicates that TgPrx1 can be used as novel vaccine antigens.

## Discussion

To develop a vaccine against *Toxoplasma gondii*, a vaccine antigen with an appropriate immune-stimulating activity is required. Recently, the use of subunit vaccines against toxoplasmosis has shown promising results [39, 40]. In our previous study, we found that TgPrx3 exerted strong immune response and conferred a protective potential against infection of mice with *T*. *gondii* PLK strain [41]. In additions, other parasitic peroxiredoxins have also been shown to be potent vaccine antigens, as reported in mouse and nonhuman primate models of cutaneous leishmaniasis induced by *Leishmania major* [42], and in mice, against microfilaria *Brugia malayi* infections [43]. In the same context, goats immunized with the recombinant peroxiredoxin of *Fasciola hepatica* displayed a 33.04% reduction in the fluke burden, and less gross and microscopic liver damage than the control group [44]. Therefore, *Toxoplasma* peroxiredoxin 1 might be an excellent candidate for the development of subunit vaccines against *T*. *gondii* infection.

To investigate the immunogenicity and protective efficacy of TgPrx1, we first detected the presence of this protein in the body fluid of infected mice to confirm its biological activity. High level of TgPrx1 was observed in the peritoneal fluid of mice experimentally infected with either RH (high-virulence strain) or PLK (low-virulence strain) during the first week of infection. The amounts of TgPrx1 detected in this study may be attributable to the in vivo growth rates of the *T*. *gondii* strains, with RH having a higher growth rate than PLK [45]. These findings suggest that TgPrx1 may be a factor in the pathogenesis of toxoplasmosis during acute infection because its release from infected cells disappears after the acute phase. Therefore, the antigenicity of TgPrx1 was markedly lower than that of TgGRA7, known to be a highly antigenic protein [34]. Previous reports have shown the ability of Prx1 to eliminate reactive oxygen species, such as hydrogen peroxide [11, 12], that are secreted by certain immune cells [46]. They are also crucial in the defense mechanisms against invading pathogens, suggesting that TgPrx1 protects *T*. *gondii* from oxidative stress derived from its hosts. In contrast, TgPrx1 may induce hyperinflammation during the acute stage of infection because triggering the production of proinflammatory cytokines, such as Il-6 and IL-12p40, by macrophages. Further research is required to clarify the role of the TgPrx1 in the virulence of *T*. *gondii*, particularly with the generation of TgPrx1-knockout parasites.

Since we demonstrated that abundant TgPrx1 is released into the body fluids during *T*. *gondii* infection, we hypothesized that it plays a role in host–parasite interactions. Importantly, TgPrx1 triggered the production of IL-6 and IL-12p40 by isolated macrophages from mouse peritoneum and RAW 264.7 cell lines. From another perspective, this reaction may support the survival of the host animal because macrophages are powerful effector immune cells that act against protozoan diseases, including *T*. *gondii* infection [5, 8]. Moreover, our data suggest that the production of IL-6 and IL-12p40 from TgPrx1-treated macrophage is NF-kB-dependent. Because previous reports indicate the role of NF-kB in modulating the host resistance against *T*. *gondii* infection [26, 47, 48], TgPrx1 is considered for a candidate of vaccine antigen.

Only one study has demonstrated the role of TgPrx1 as an immunomodulator using bone marrow-derived macrophages, and revealed that it primarily activates the alternative pathway and mainly induces the secretion of the anti-inflammatory cytokine IL-10. However, a reduction in IL-1β, but no significant effect on IL-12p40, has also been reported [13]. This discrepancy might be attributable to the use of different parasite strains, different expression system and concentrations of recombinant proteins, and macrophages of different origins. In the present study, we investigated the immune response of peritoneal macrophages treated with TgPrx1 because this type of cells provides an important advantage than bone marrow-derived macrophages. In addition to the full maturation, the primed and sensitized condition of macrophages harvested from the peritoneal cavity of mice after injection with thioglycollate renders them more similar to the normal status of host immune response under stimulation of infectious or other immune-stimulating agents [49]. Moreover, thioglycollate-elicited macrophages collected from mouse peritoneal cavity exerte a professional role against *T*. *gondii* especially for cytokine production [6, 50–52]. Although both peritoneal macrophages and bone marrow-derived macrophages are frequently used for the evaluation of basic, essential mechanisms of immune recognition and engagement of an immune response, they are not producing the same mediators upon the same conditions because the kinetics are not be the same and both populations are not sharing some common cytokine/chemokine receptors. For example, peritoneal macrophages were expressing more MHC class II and CD86 molecules rather than bone marrow-derived macrophages which express significantly higher level of CD80 and CD115. Therefore, the levels of MHC class II in peritoneal macrophages may be highly incorporated in inflammatory cytokine production [49, 53–54].

Based on our previous results and successful trials of *T*. *gondii*-derived enzymes as vaccine candidates, such as protein disulfide isomerase [55], calcium-dependent protein kinase-3 [56], and glutathione reductase [57] and peroxiredoxin 3 [41], we investigated the protective efficacy of TgPrx1. Interestingly, mice immunized with TgPrx1 displayed significant resistance to *T*. *gondii* infection suggesting the considerable immunoprophylactic potency. Our study showed that TgPrx1 as an effective stimulator of macrophages and spleen cells for production of IL-12 and IFN-γ, respectively. Th1 immune cells such as macrophages, CD4^+^ and CD8^+^ T cells are predominantly triggered by the IL-12 and result in significant production of IFN-γ, which stimulates effector cells for pathogen killing [58]. Moreover, TgPrx1 more potently induced specific IgG1 antibodies than the specific IgG2c antibodies. Furthermore, the contribution of specific anti-TgPrx1 antibodies in the TgPrx1-induced protection was confirmed because the mice immunized with these antibodies survived for a longer time compared with the control group inoculated with control rabbit antibodies (S1 Fig). Thus, the ability of TgPrx1 is to stimulate both humoral and cell-mediated immunity against *T*. *gondii* infection. Although the immune-stimulating responses of TgPrx3 were higher than those of TgPrx1, the protective properties of TgPrx1 were higher compared with TgPrx3 [41]. This was evidenced in the levels of IL-12p40 production from peritoneal macrophages and the production of the specific IgG1 and IgG2c antibodies after immunization. These results indicated that an appropriate level of immune response would be required for successful protection against *T*. *gondii* infection. These immune responses induced by the naked recombinant TgPrx1 alone consistent with those in several reports revealed successful vaccine antigens against *T*. *gondii* infection [59–62]. Therefore, our future studies will focus on the combination of this protein with a potent adjuvant to improve its protective efficacy against lethal toxoplasmosis.

## Conclusion

In the present study, we found that TgPrx1 possessed immune-stimulating activity. Moreover, immunization with TgPrx1 induced antigen-specific humoral and cellular immunity and controlled *T*. *gondii* infection in mice. Thus, TgPrx1 may indicate its candidacy as a potent vaccine against *T*. *gondii* infection and toxoplasmosis.

## Supporting information

S1 Fig Administration of *T*. *gondii*-infected SCID mice with anti-TgPrx1rabbit IgG antibodies. The protective effect of purified antibodies was tested by inoculating of SCID mice with anti-TgPrx1 rabbit IgG antibodies (n = 6) and control rabbit IgG antibodies (n = 5) by an amount of 1 mg prepared in 500 μL of PBS. The mice were immunized with the IgG antibodies via the intraperitoneal route each other day starting from 1 day prior until 9 day post infection (dpi) with 10^3^ PLK tachyzoites. Survival of mice was checked twice a day until all mice were succumbed. Survival curves were generated with the Kaplan–Meier method. According to the log-rank test, the differences among the two groups were significant (*P < 0.05).
(PDF)
