# Supplementary material for: Immunization with Toxoplasma gondii peroxiredoxin 1 induces protective immunity against toxoplasmosis in mice
Source: PLoS One. 2017 Apr 27;12(4):e0176324. doi: 10.1371/journal.pone.0176324 (PMC5407612; doi:10.1371/journal.pone.0176324)
Supplement: S1 Fig — The protective effect of purified antibodies was tested by inoculating of SCID mice with anti-TgPrx1 rabbit IgG antibodies (n = 6) and control rabbit IgG antibodies (n = 5) by an amount of 1 mg prepared in 500 μL of PBS. The mice were immunized with the IgG antibodies via the intraperitoneal route each other day starting from 1 day prior until 9 day post infection (dpi) with 103 PLK tachyzoites. Survival of mice was checked twice a day until all mice were succumbed. Survival curves were generated with the Kaplan–Meier method. According to the log-rank test, the differences among the two groups were significant (*P < 0.05). (PDF) [file pone.0176324.s001.pdf]

Supporting Information

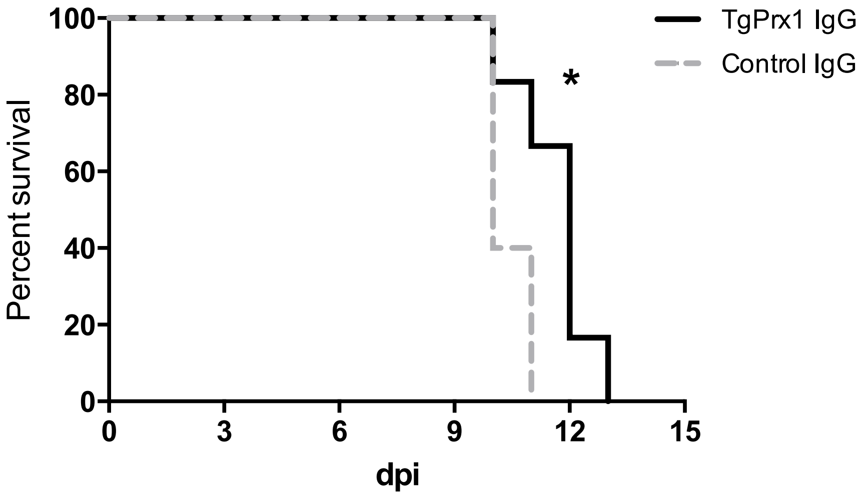

S1. Fig. Administration of *T. gondii*-infected SCID mice with anti-TgPrx1rabbit IgG antibodies. The protective effect of purified antibodies was tested by inoculating of SCID mice with anti-TgPrx1 rabbit IgG antibodies (n=6) and control rabbit IgG antibodies (n=5) by an amount of 1 mg prepared in 500 μL of PBS. The mice were immunized with the IgG antibodies via the intraperitoneal route each other day starting from 1 day prior until 9 day post infection (dpi) with 10^3 PLK tachyzoites. Survival of mice was checked twice a day until all mice were succumbed. Survival curves were generated with the Kaplan–Meier method. According to the log-rank test, the differences among the four groups were significant (*$P < 0.05$).
